# Prevalence and characterization of severe asthma in Hungary

**DOI:** 10.1038/s41598-020-66445-4

**Published:** 2020-06-09

**Authors:** Zsuzsanna Csoma, Zsófia Gál, András Gézsi, Irén Herjavecz, Csaba Szalai

**Affiliations:** 1 National Korányi Institute of Pulmonology, Budapest, Hungary; 2 Department of Genetics, Cell- and Immunobiology, Semmelweis University, Budapest, Hungary; 3 Department of Measurement and Information Systems, Budapest University of Technology and Economics, Budapest, Hungary; 4 Heim Pál Pediatric Hospital, Budapest, Hungary

**Keywords:** Respiratory tract diseases, Asthma

## Abstract

Background: Severe asthma (SA) database was established in Hungary to estimate the prevalence of SA and to define and analyze clinical phenotypes of the patients. Methods: SA questionnaires were sent out to 143 public pulmonary dispensaries specialized for diagnosing and caring pulmonary patients. Data of 520 SA patients were evaluated. Results: The prevalence of SA within the asthmatic population in Hungary was 0.89%. The mean age of patients were 56.4 ± 13.4 years, SA were more frequent in females (64%), the prevalence of allergy was 56.6%, 72.1% of patients had persistent airflow limitation (FEV1 < 80%), 37.9% severe airway obstruction (FEV1 ≤ 60%), 33.6% required systemic corticosteroid maintenance therapy, 21.5% had salicylate intolerance and 43.2% rhinosinusitis. A Bayesian dependency network was calculated which revealed several interdependencies between the characteristics. E.g. there was a strong association between salicylate intolerance and rhinosinusitis, more patients received regular systemic corticosteroid treatment who had salicylate intolerance and the proportion of salicylate intolerance was significantly higher in females. Conclusion: The prevalence of SA was determined in Hungary which was lower than in other studies. Using a Bayesian-based network analysis several interdependencies were revealed between patient characteristics.

## Introduction

Severe asthma (SA) accounts for only a small percentage of patients with asthma, but the care of these patients accounts for a considerable portion of the health care costs posed by asthma. In addition, SA was found to be associated with impaired quality of life and a higher risk of severe exacerbations potentially leading to death compared to non-SA^1,2^.

Despite the intensive research focused on the field of SA, many aspects of this disease remained unclear. Data on the prevalence of SA are quite heterogeneous, and phenotypes have only been partly characterized. Studying large number of these patients is necessary, as researchers generally agree that asthma, including also SA, can be defined as a syndrome, in which an appreciable overlap exists in phenotypes, but there are different etiologic factors, inflammatory processes in the background and there is also a variance in prognosis and treatment sensitivity^3,4^.

In recent years, several European and North American consortia were established with the aim at improving our understanding of the mechanisms of SA^5–9^. Such studies are necessary for a better recognition of SA as a heterogeneous condition comprising multiple phenotypes which may have prognostic value and therapeutic implications.

To understand SA better, a standardized definition is indispensable^3^. SA is defined by the European Respiratory Society/American Thoracic Society (ERS/ATS) as asthma requiring treatment with high-dose inhaled corticosteroids plus a second controller with or without systemic corticosteroids to maintain control of the disease or, despite this therapy, having sub-optimally controlled asthma^10^. According to the recommendation of Global Initiative for Asthma (GINA) in 2019, SA is a subset of difficult-to-treat asthma. Difficult-to-treat asthma is considered when the disease remains uncontrolled despite the previously mentioned high dose preventer treatment. Contributory factors may include incorrect diagnosis, incorrect inhaler technique, poor adherence, and comorbidities, like rhinosinusitis, salicylate sensitivity and gastroesophageal reflux disease. SA is now defined as asthma remains uncontrolled despite maximal optimized therapy and treatment of contributory factors, or that worsens when high dose treatment is decreased, i.e. relatively refractory to corticosteroids^11^.

Depending on the study definition, and owing to the differences in the environment, health care system and expertise of the treatment team the range of published prevalence data is between 2.5% and 20% with an estimated average value about 5–10% of all asthmatics^12–17^. We have not had accurate data about the Hungarian prevalence of SA. Therefore, we built up a SA patient database involving patients who have been cared in the pulmonary network in Hungary and met the ERS/ATS definition of severe refractory asthma. Our aims were to determine SA prevalence in Hungary, to further define and analyze clinical phenotypes in subjects with well characterized SA and to find interdependencies between patient characteristics collected through the questionnaires.

The present study describes the results of this survey and the main clinical features of severe asthmatics registered over the country.

## Methods

There are 156 public pulmonary outpatient clinics in Hungary, called pulmonary dispensaries, which are specialized for diagnosing and caring pulmonary patients. They provide data annually on incidence and prevalence of asthma, chronic obstructive lung disease (COPD), tuberculosis (TB) and lung cancer through an online system. Data were analyzed in the methodological center of National Korányi Institute of Pulmonology (NKI) every year. The number of treated severe asthma patients has been provided separately since 2009 in this system, the presented data is from 2011. The following definition was used: “Asthma remaining uncontrolled despite high dose inhaled corticosteroids (>800 µg budesonide equivalent/day) in a combination with long-acting β2-agonists and/or additional controller therapy, such as theophylline and/or leukotriene modifiers accidentally. Uncontrolled was defined as persistent symptoms or recurrent exacerbations (≥4/year) requiring frequent or continuous systemic steroid treatment.”

In line with the data collection, we also started a severe asthma survey to confirm the validity of these data. The survey was a special severe asthma questionnaire regarding information of age, gender, disease onset and duration, lung function, atopy, smoking habits, systemic steroid claim, exacerbations and salicylate/non-steroid anti-inflammatory drug (NSAID) intolerance (Supplementary Table 1). Questionnaires along with study design and written informed consents were sent to the public pulmonary dispensaries. All data were provided by clinicians specialized for chest diseases.

Data of the returned questionnaires from the dispensaries (group 1) were analyzed further and the results were compared to the similar data of 104 well-defined severe asthma patients who had been cared in the asthma outpatient clinic of NKI which served as a methodological center (group 2). This latter group operated as a reference group to verify the reliability of questionnaire data received from the dispensaries. Patients were all diagnosed with severe asthma by a specialist and had been treated for at least one year, but in most of cases for many years according to GINA guidelines.

The study was conducted in accordance with the Ethical Guidelines for Biomedical Research Involving Human Subjects, the ethical principles of the Declaration of Helsinki and was approved by the Scientific and Research Ethics Committee of the Medical Research Council (Ministry of Health) and the local ethics committee of the NKI (number: 8–375/2009-1018EKU). Informed written consent was obtained from each patient.

### Statistical analysis

Continuous variables are presented as means ± SD, and categorical variables are presented as both number and percentage. Mann-Whitney and t-test were used to compare continuous variables between groups. Categorical variables were compared using chi-square (χ^2^) test. Pearson’s correlation analysis was used to analyze variable correlations, p<0.05 was considered statistically significant. Analyses were performed using the GraphPad Prism Software Version 5.0 and Medcalc on line calculator^18,19^.

For the calculation of direct causal relevance between patient characteristics a Bayesian statistical framework was used, developed by our research group, named Bayesian network based Bayesian multilevel analysis of relevance (BN-BMLA). Detailed description of the method can be found elsewhere^20–22^. Within the Bayesian statistical framework the uncertainty of the validity of a discrete hypothesis is expressed by a probability, which is interpreted as an a posteriori belief in the hypothesis. The a posteriori probability of the strong relevance is between 0 and 1, where 1 means that the two variables most certainly have a dependency relationship with each other, on the other hand 0 means that there is no such relationship. A posteriori probabilities of strong relevance greater than 0.75 are regarded as convincing, values between 0.5 and 0.75 indicate suggestive relevance^20^.

## Results

### Prevalence of severe asthma within the asthmatic population

The number of asthma patients, who were qualified as SA according to the study definition in all pulmonary dispensaries was 2,434 over the country. This number is the result of the corrections based on the experiences of the questionnaires sent back to the methodological center (see below). The number of total asthma population, which has been registered in the same system annually was 272,883. It means that the prevalence of SA within the asthmatic population in Hungary was 0.89%.

### Comparison of data of SA patients from public dispensaries and methodological center

143 of 156 public pulmonary dispensaries participated in the SA survey study. Thirteen of them do not treat severe asthma patients at all. Altogether 48% of participating dispensaries sent completed questionnaires back, which resulted in data of 519 patients.

Nineteen percent of the 519 patients were excluded from further analysis because clinical features of these patients were not undoubtedly met the ATS/ERS criteria for severe refractory asthma. We excluded smokers (≥5 pack year tobacco use) and patients with very late-onset disease (age at disease onset ≥65 years old). We also excluded patients from further analysis with normal lung function who had no recurrent exacerbations requiring frequent or continuous systemic steroid treatment in their medical history.

Following the exclusions, data of 416 patients from the public dispensaries were evaluated (group 1) and compared with data of 104 severe asthma patients (group 2) registered in the asthma outpatient clinic of NKI (group 2) which served as a methodological center. In this way, altogether data of 520 SA patients were evaluated. The data of all patients are presented in Supplementary Table 2. The characteristics and comparison of the two groups along with the combined data are presented in Table 1.

**Table 1:** Clinical characteristics and comparisons of severe asthma patients.

Characteristics	Total	Group 1	Group 2	p-value*
Number	520	416	104	—
Age (years)	56.4 ± 13.4	57.5 ± 13.2	52.0 ± 13.3	0.0002
Disease duration (years)	22.2 ± 12.5	21.5 ± 11.8	24.9 ± 14.6	0.01
FEV1, personal best (% pred.)	67.3 ± 19.5	66.6 ± 20.5	70.0 ± 17.2	0.12
Worst FEV1 (% pred)	39.3 ± 13.5	38.7 ± 13.3	41.7 ± 14.0	0.05
Gender (male/female) (male%)	186/330 (36.0)	151/261 (36.6)	35/69 (33.7)	0.57
Disease onset (childhood (<12 y)/adult) (childhood%)	65/453 (12.4)	37/377 (8.9)	28/76 (26.9)	<0.0001
Allergy yes/no (yes%)	287/220 (56.6)	233/170 (57.8)	54/50 (51.9)	0.27
Systemic corticosteroid dependence yes/no (yes%)	175/345 (33.6)	144/272 (34.6)	31/73 (29.8)	0.34
Corticosteroid “burst” yes/no (yes%)	414/105 (79.8)	332/83 (80.0)	82/22 (78.8)	0.82
Salicylate intolerance yes /no (yes%)	111/406 (21.5)	70/343 (17.0)	41/63 (39.4)	<0.0001
Rhinosinusitis yes/no (yes%)	223/293 (43.2)	136/277 (32.9)	87/16 (84.4)	<0.0001

Clinical characteristics and comparisons of severe asthma patients registered in the pulmonary dispensary network (group 1) and in the outpatient clinic of NKI (group 2). Combined data of these two groups are also presented (total). Continuous variables are presented as means ± SD, and categorical variables are presented both as numbers and percentage.

*Group1 vs group2; Comparisons of means and proportions were carried out by Medcalc online calculator^18^.

There was no difference between the groups in gender distribution, prevalence of allergy, systemic corticosteroid dependence, corticosteroid burst treatment and the mean value of personal best FEV1. Although the mean worst FEV1 values were statistically different, the difference was clinically not relevant (38.7% vs 41.7% in group 1 vs 2, respectively)

On the other hand, as can be seen in Table 1, there were significant differences between the two groups in many respects. Average age of the patients was lower in group 1, while the duration of asthma was shorter in the dispensary group (p = 0.0002 and 0.01, respectively). Significantly more patients were in group 2, who developed asthma in childhood (age < 12; 8.9% vs. 26.9% p < 0.0001), and there were significantly more patients in group 2 with salicylate/NSAID intolerance (17.0% vs 39.4%; p < 0.0001) and rhinosinusitis (32.9% vs. 84.4%; p < 0.0001).

### Clinical features of severe asthmatics registered over the country based on the questionnaire

Analyzing the functional data of the entire study population showed that 72.1% of severe asthma subjects had persistent airflow limitation, defined as FEV_1_ <80% (mean ± SD: 58.1 ± 13.6% /of predictive), and in this regard there was no difference between the groups (72.1% for both groups). Severe airway obstruction, defined as FEV_1_ ≤ 60% (of predictive), was in 37.9% of all severe asthmatics, with a mean FEV_1_ value of 47.5 ± 9.5%. In the remainder 62.1% of all patients the personal best FEV_1_ was above 60% (mean 79.3 ± 13.8%). There was no correlation between the personal best FEV_1_ and asthma duration.

Among patients where the disease started in childhood there were significantly more allergic than among patients with adult-onset asthma (80.9% vs 53.4%; p < 0.0001, in childhood-onset vs adult-onset, respectively).

There was no difference in the proportion of patients with severe airway obstruction (best FEV1 < 60) between allergic and non-allergic severe asthmatics (34.1% vs 32.3%, respectively, ns).

**Figure 1:**
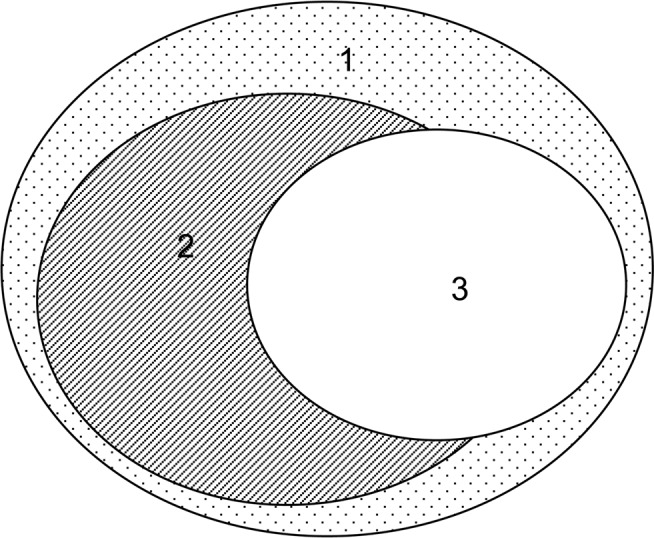
Proportion of severe asthma patients with allergy and allergic/non-allergic severe asthmatics with systemic corticosteroid dependence. The areas of the ellipses in the Venn diagram represent the percentage of the investigated patient groups and the placement of them shows the relationship and the overlap of these data sets. 1. Total asthma population (n = 520). 2. Proportion of patients with allergy: 56.6%. 3. Proportion of patients with systemic corticosteroid dependence: 33.6% from which 58.0% is allergic and 42.0% non-allergic).

We could not find any relationship between systemic steroid dependence and non-allergic severe asthma as the ratio of allergic/non-allergic disease was similar in cases requiring maintenance systemic steroid treatment and in cases without that (100 allergic/92 non-allergic vs 185 allergic/125 non-allergic, 58.0/42.0% vs 55.9/44.1%, ns, with and without systemic steroid treatment, respectively) (see also in Fig. 1).

### Interdependencies between characteristics of patients

**Figure 2:**
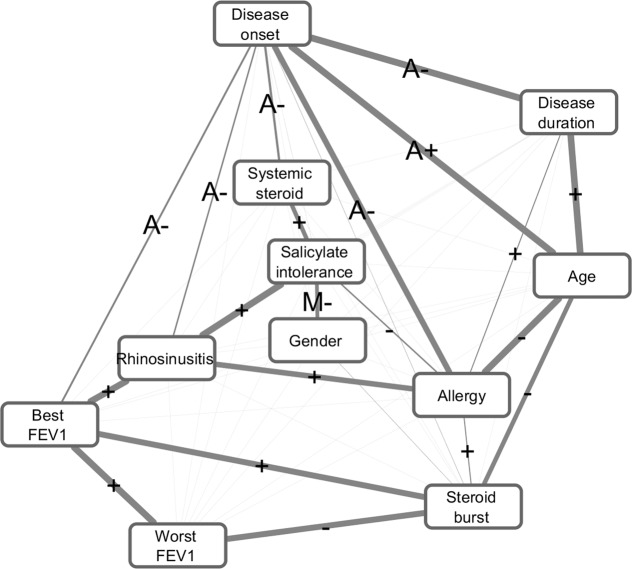
Bayesian dependency network of 10 characteristics in 520 severe asthmatic patients. An edge between two nodes (i.e. characteristics) represents direct casual relevance, and its width is proportional to the probability of the corresponding nodes being directly relevant to each other (i.e. taking into account both possible edge directions assuming an underlying Bayesian network). Those connections are depicted where the a posteriori values between characteristics are larger than 0.15. The exact values can also be seen in Table 2. Positive (+) sign on an edge means that the direction of the relation is positive, e.g. patients with higher “best FEV1” have higher chance of having better “worst FEV1” value. Negative (−) sign indicates that the direction of the relation is opposite, “A” indicates adult, “M” male. E.g. “A-“ on the edge between “Disease onset” and “Allergy” means that allergy associates with childhood onset and not with adult onset. These relations can be seen in Fig. 3 and Table 3.

For the calculation of direct causal relevance between patient characteristics, a Bayesian statistical framework was used, named Bayesian network based Bayesian multilevel analysis of relevance (BN-BMLA)^20–22^. Altogether 10 characteristics collected through the questionnaires were involved in this analysis and all the 520 patients were included. The calculated Bayesian dependency network can be seen in Fig. 2. The network is an undirected graph where an edge between two nodes (here characteristics) represents direct casual relevance (i.e. a node is a direct cause of the other node), and its width is proportional to the probability of the corresponding nodes being directly relevant to each other. Those connections are depicted where the a posteriori values between characteristics are larger than 0.15, and these also can be seen in Table 2.

**Table 2:** A posteriori probability of directed causal relevance between characteristics of patients calculated by BN-BMLA.

Characteristic 1	Characteristic 2	A posteriori probability of direct causal relevance between Characteristic 1 and Characteristic 2
Allergy	Disease duration	0.28
Allergy	Disease onset	0.90
Allergy	Age	1.0
Allergy	Rhinosinusitis	0.86
Allergy	Salicylate intolerance	0.33
Allergy	Steroid burst	0.28
Disease duration	Disease onset	1.0
Disease duration	Age	1.0
Disease onset	Age	1.0
Disease onset	Best FEV1	0.40
Disease onset	Systemic steroid	0.44
Disease onset	Rhinosinusitis	0.34
Age	Steroid burst	0.73
Best FEV1	Worst FEV1	1.0
Best FEV1	Rhinosinusitis	0.98
Best FEV1	Steroid burst	0.91
Worst FEV1	Steroid burst	0.92
Gender	Salicylate intolerance	0.63
Systemic steroid	Salicylate intolerance	0.71
Rhinosinusitis	Salicylate intolerance	1.0

**Figure 3:**
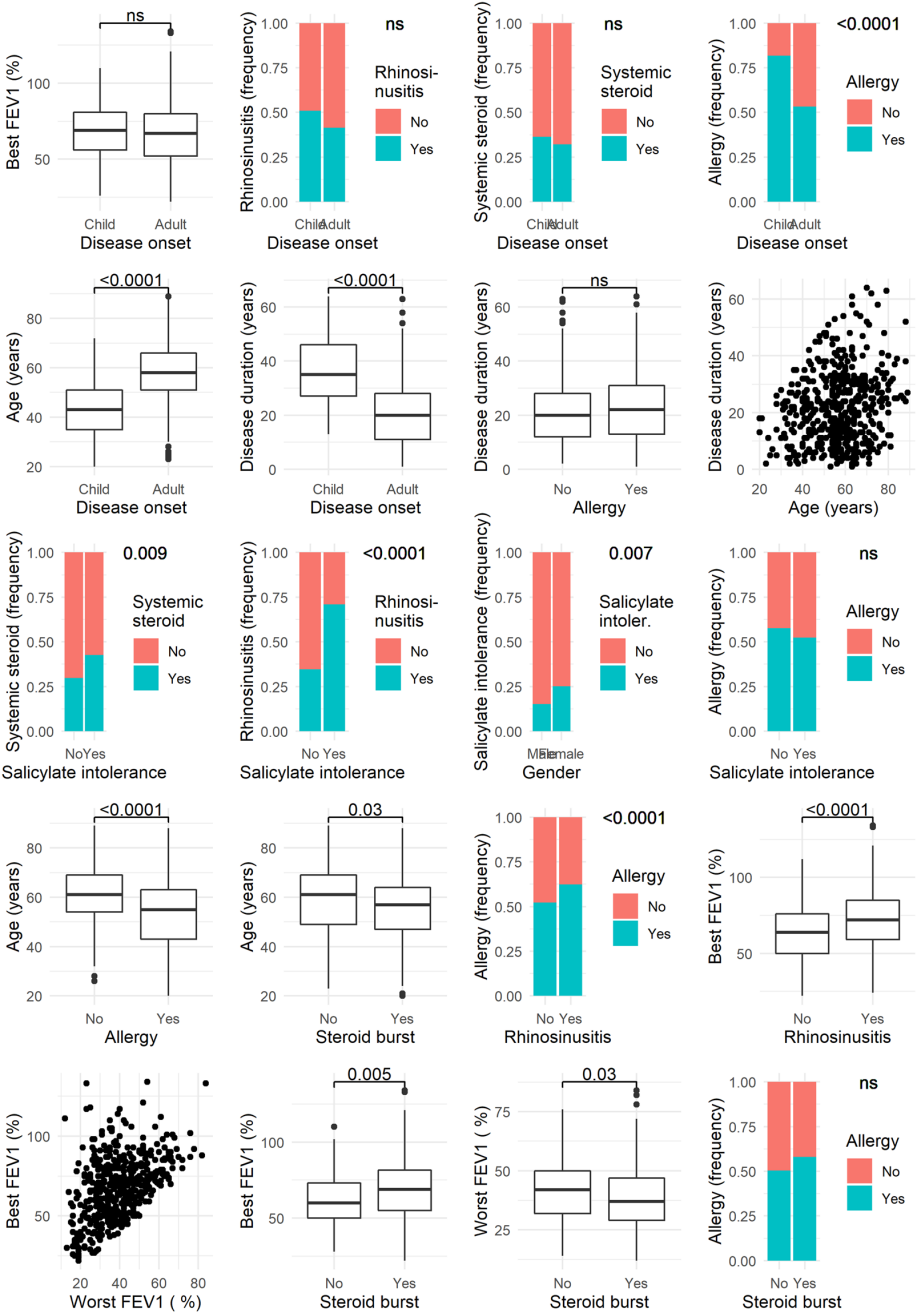
Pairwise relations of some characteristics. Only those relations are shown where the a posteriori probability of direct casual relevance is higher than 0.15 and there is an edge between them in Fig. 2. The a posteriori probability values are shown in Table 2. The relevance revealed by BN-BMLA were confirmed with conventional statistics. Comparisons of means and proportions were carried out by Medcalc online calculator^18^. P values indicating significant differences (p < 0.05) are shown for each comparison, ns means non-significant. Values and significant results are also shown in Table 3.

A posteriori probability of directed causal relevance between characteristics of patients calculated by BN-BMLA and depicted in Figs. 2 and 3. Values greater than 0.75 are regarded as convincing, values between 0.5 and 0.75 indicate suggestive relevance^20^. The directions of the dependencies can be seen in Fig. 2. Disease onset differentiates whether the asthma started in childhood or in adulthood using age 12 years as a cut off.

This analysis confirmed some evident or previously shown dependencies, e.g. there was a strong connection between allergy and rhinosinusitis with an a posteriori value of (P = 0.86), between disease onset (childhood or adult) and allergy (P = 0.9), between best and worst FEV1 and also showed some novel ones.

E.g. there was a strong direct positive casual relevance between salicylate intolerance and rhinosinusitis (P = 1), best FEV1 value and rhinosinusitis (P = 1) and interestingly between best FEV1 and steroid burst therapy (P = 0.91). This latter means that within the SA population the chance of oral steroid burst therapy increased with better lung functions when best FEV1 were considered. The connection was inverse in case of worst FEV1 and oral steroid burst therapy (P = 0.92). There was a suggestive relevance between gender and salicylate intolerance (P = 0.63). In this case ‘M-‘ in the Fig. 2 on the edge between the two nodes means that the prevalence of this characteristics was lower in males. There was also a suggestive relevance between oral systemic steroid treatment and salicylate intolerance (P = 0.71), and age and oral steroid burst therapy (P = 0.73).

The data behind these calculations are shown in Fig. 3, where pairwise relations of these characteristics are depicted. The relevance revealed by BN-BMLA were confirmed with conventional statistics. As can be seen in Table 3, all the above-mentioned connections proved to be statistically significant. E.g. SA patients with rhinosinusitis had on average higher best FEV1 than patients without this comorbidity (72.4 ± 20.0% vs. 63.2 ± 18.8%; p < 0.0001). The mean age of patients who received corticosteroid burst therapy was younger (55.8 ± 13.0 vs. 59.0 ± 14.9 years; p = 0.03). Significantly more patients received regular oral systemic corticosteroid treatment who had salicylate intolerance (44.1% vs. 30.8%; p = 0.009), the proportion of rhinosinusitis was significantly higher in patients with salicylate intolerance (72.0% vs. 34.7%; p < 0.0001; the proportion of rhinosinusitis with and without salicylate intolerance, respectively), the proportion of salicylate intolerance was significantly higher in patients with rhinosinusitis (36.2% vs. 10.3%; p < 0.0001) and the proportion of salicylate intolerance was significantly higher in females (25.3% vs. 15.1%; p = 0.007; proportion of salicylate intolerance in females and males, respectively) (Table 3). The group1 and group2 did not differ in these respects.

**Table 3:** Evaluation of some more interesting dependencies revealed by BN-BMLA (Figs. 2 and 3, Table 2).

Characteristics	Values	p-value
Prevalence of allergy in adult onset asthma (%)	53.4	
Prevalence of allergy in childhood onset asthma (%)	80.9	<0.0001
Mean age if allergy yes (years)	53.0 ± 12.5	
Mean age if allergy no (years)	61.0 ± 12.3	<0.0001
Mean age if steroid burst yes (years)	55.8 ± 13.0	
Mean age if steroid burst no (years)	59.0 ± 14.9	0.03
Mean best FEV1 if rhinosinusitis yes (%)	72.4 ± 20.0	
Mean best FEV1 if rhinosinusitis no (%)	63.2 ± 18.8	<0.0001
Mean best FEV1 if steroid burst yes (%)	68.5 ± 20.5	
Mean best FEV1 if steroid burst no (%)	62.4 ± 16.7	0.005
Mean worst FEV1 if steroid burst yes (%)	38.6 ± 13.5	
Mean worst FEV1 if steroid burst no (%)	41.8 ± 13.2	0.03
Proportion of males with salicylate intolerance (%)	15.1	
Proportion of females with salicylate intolerance (%)	25.3	0.007
Proportion of systemic corticosteroid dependence in patients with salicylate intolerance (%)	44.1	0.009
Proportion of systemic corticosteroid dependence in patients with no salicylate intolerance (%)	30.8	
Proportion of salicylate intolerance in patients with systemic corticosteroid dependence (%)	28.2	0.008
Proportion of salicylate intolerance in patients with no systemic corticosteroid dependence (%)	18.1	
Proportion of rhinosinusitis in patients with salicylate intolerance (%)	72.0	<0.0001
Proportion of rhinosinusitis in patients with no salicylate intolerance (%)	34.7	
Proportion of salicylate intolerance in patients with rhinosinusitis (%)	36.2	<0.0001
Proportion of salicylate intolerance in patients without rhinosinusitis (%)	10.3	

Comparisons of means and proportions were carried out by Medcalc online calculator^18^.

## Discussion

The primary objective of the study was to standardize the definition of severe asthma in Hungary. Furthermore, to record epidemiologic data and to set up a reliable national clinical registry of patients with severe asthma, which underlies a basis for deeper analysis of phenotypes and endotypes of this disease.

To estimate the prevalence of severe asthma in Hungary, NKI, as a methodological center, asked the pulmonary dispensaries to report the number of asthma patients who fulfill the ATS criteria of severe refractory asthma. This determination essentially covers asthma patients treated according to the fourth and fifth step of GINA asthma treatment guidelines^23,24^. The reported percentage of severe asthma in the total asthma population was 0.89%, which is lower than the prevalence reported in most other studies^12–16^. However, there is a considerable variation in the prevalence estimates of severe asthma in different countries ranging from 2.5% to 8.1%, even some studies reported as high as 20%, indicating partly population heterogeneity, different environments and health care system, but also support the notion that current guidelines may be ambiguous^12–17^.

It must be noted that because only 48% of the pulmonary dispensaries sent back the questionnaires, the real prevalence of SA could be different from the calculated value. It must be added, however, that besides questionnaires, there are continuous and active communications on different platforms among pulmonologists treating asthma patients, and the disease definition was unified all over the different asthma centers. The 2,434 SA and the 272,883 total asthma populations were the registered patients in all pulmonary dispensaries and these numbers were the results of the corrections based on the experiences of the questionnaires sent back to the methodological center. In Hungary, a well-organized network for caring patients with lung diseases gives a unique opportunity to carry out survey studies, although some patients including severe asthmatics are cared in outpatient clinics of hospital centers.

Data obtained from pulmonary dispensaries were considered to be reliable as we did not find significant differences in the most important characteristics between the two study groups. Thus, there was a similarity in the gender (male/female) distribution − with a clear predominance of females in both groups −, functional status of patients, percentage of atopic cases and rate of patients requiring maintenance systemic corticosteroid therapy and the majority of the disease was adult-onset in both groups.

The mean age of patients in pulmonary dispensaries was higher than in the center. This may raise the problem that patients with COPD were included in the dispensary group^25^. To avoid this, we excluded smokers and patients with very late-onset disease. It also reduced the possibility that COPD patients were included, that the average duration of disease was 21.5 (±11.8) years in this study group, which means that severe obstructive airway disease began at the age of 36 years on average.

The mean age of all the patients (56.4 ± 13.4 years) was similar than it had been reported in most international studies, although in some countries it was significantly lower (e.g. in Germany 44.4 ± 20.4) or higher (e.g. in Netherlands 62.5 ± 16.5)^12–17^.

Severe asthma started predominantly in adulthood (using age 12 as a cut off) in both study groups, but the proportion of patients with an adult-onset disease was higher in pulmonary dispensaries (91.1% vs 73.1%). The explanation for this may be the fact that children with severe asthma are usually cared in a few hospital centers in Hungary. Pulmonary dispensary network was for care of tuberculosis originally, and nowadays adult patients with full spectrum of lung diseases are treated there. Therefore, adolescents with severe asthma are usually directed from special pediatric asthma clinics to adult centers specialized for treatment of asthma, like the NKI, explaining the higher proportion of patients with early-onset disease in group 2.

The higher prevalence of the adult-onset disease, which was also found in other studies, supports the observation that this phenotype of asthma is often more severe than early-onset asthma^17,26^.

There were also significant differences in the prevalence of salicylate/NSAID intolerance and rhinosinusitis between the dispensary group and patients treated in the center, with higher values in the latter group.

The difference in rhinosinusitis (84.4% vs 32.9%) was primarily seen as a problem of interpretation. Nevertheless, even the rate of severe sinusitis requiring ear, nose and throat (ENT) interventions was proven to be higher (51%) in severe asthma patients of the center than the total proportion of rhinosinusitis indicated in the dispensary group. Due to the differences in the criteria for upper airway diseases in the relevant international consortia, data are difficult to compare. The prevalence of sinusitis was 67% in the SARP study, while severe cases, requiring interventions by an ENT specialist occurred in 27% (6, 40)^8,27^. In a Belgian SA study the prevalence of rhinosinusitis was 49%^28^.

Salicylate/NSAID intolerance was detected in the center in a significantly higher proportion (39.4% vs 17.0%) when compared to dispensaries. The strong connection of salicylate intolerance and rhinosinusitis together with asthma, which can also be seen in Fig. 2 and Table 3, known as Samter’s Triad, is a well-known triad of symptoms^29^, although this phenomenon in the SA populations has not yet been published. As there were significantly more patients with rhinosinusitis in group 2, the Samter’s Triad might also explain why significantly more patients with salicylate intolerance were in this group. The percentage of non-allergic asthma, with which polyposis and salicylate/NSAID intolerance are so often associated, was similar in the two groups. However, it is also probable that the higher proportion of salicylate/NSAID intolerance among our patients is at least partly a consequence of the fact that this subject in the center has been investigated since the 1980s and the question of possible hypersensitivity is assumed for each patient, so it is an important item of their medical history^30^. Significant differences can also be found between countries in the ERS SHARP severe asthma registries where the prevalence of salicylate intolerance ranged between 8.2 and 26.9%^17^.

In the light of international data, the predominance of female gender is a well-known phenomenon in severe asthma. The relative proportion of female patients was 64% in the entire severe asthma database in Hungary, which is similar in most countries^8,17,31^. A possible explanation of this phenomenon came from a mouse study where testosterone decreased type 2 and IL-17A inflammation^32^. In addition in humans, women had an increased number of circulating group 2 innate lymphoid cells compared to men^33^. These lymphocytes play a central role in the propagation of allergic responses and asthma.

We analyzed the documented personal best FEV_1_ values (expressed in the percentage of predictive value) of functional parameters, although in the majority of studies the postbronchodilator FEV_1_ was evaluated. Considering that in our study data collection was performed, and in most cases, patients were not assessed directly, we supposed that physicians caring these subjects regularly, more likely have reliable data on personal best values. The mean value of personal best FEV_1_ was 67.3% in the entire database, which was not significantly different from prebronchodilator values reported in international studies^5,6,8,17,31^.

When analyzing the data of the entire study population further, we found that 72.1% of severe asthma subjects had persistent airflow limitation (FEV_1_<80%), but severe airway obstruction was detected only in 37.9% of cases, with a 47.5% personal best FEV_1_ mean value. In the TENOR study, the ratio of severe fixed airway obstruction, defined as postbronchodilator FEV_1_ below 60%, was 27.8%, which was calculated for the entire study population with moderate and severe cases in an equal proportion^6^. In the SARP study, 80% of severe asthma subjects had persistent airflow obstruction defined as FEV_1_<80% and nearly half of them had a baseline FEV_1_<60% when bronchodilators were withhold before spirometry.

The percentage of allergic cases was 56.6% in the entire population of our study which was similar to the results of severe asthma consortia, as the rate of allergy confirmed by skin-prick testing was 58% in the ENFUMOSA, 71% in the SARP studies^5,8^. In the British Registry, the co-occurrence of RAST and skin test positivity was 59.2% in severe asthmatics^31^.

In our population the prevalence of allergy was significantly higher in patients with childhood-onset (using age 12 as a cut off) than in patients with adult-onset asthma (80.9% vs 53.4%). This confirms previous observations that early-onset disease is distinctively associated with a more atopic and allergic conditions^34–36^.

The rate of severe asthmatics requiring systemic corticosteroid maintenance therapy was 33.6% in our study. In this respect, significant differences could be found between countries in the ERS SHARP study where the rate of systemic corticosteroid maintenance therapy ranged between 10.2% (Spain) and 71.9% (Italy) suggesting different prescribing regimens across Europe^17^.

The BN-BMLA analysis revealed interdependencies between the 10 characteristics collected through the questionnaires from the 520 patients. One of the advantages of this statistical method that it evaluates the data as a system without preconception, thus it can reveal less evident relations which are often dismissed and not analyzed using conventional statistical methods. E.g. the majority of SA studies has not studied (at least not published) the proportions of genders in salicylate intolerance. The phenomenon that the salicylate intolerance is significantly higher in females has only been published as an ERS International Congress abstract from the WATCH difficult asthma study^19^. In an earlier European survey it was also found that in aspirin intolerant asthma females outnumbered males, the onset of symptoms occurred significantly earlier and the disease was more progressive and severe than in males^37^.

In the SARP study where patients with severe, moderate, and mild asthma were compared, sinusitis was found to be associated with SA and in this way lower lung function^8^. In our study population, the prevalence of rhinosinusitis was also very high, but interestingly associated with a better lung function within the SA patients. This relation in SA patients has not been investigated (or not published) in other studies. According to these data and the results of a Japanese study^14^, where chronic paranasal sinusitis was associated with uncontrolled asthma within the SA group, it can be hypothesized that rhinosinusitis might increase asthma severity by impairing the controllability of asthma. In this way patients with this comorbidity even with better lung functions might have a higher chance to develop SA.

It is also an interesting finding that SA patients with better “best FEV1” values had on average a higher chance of steroid burst therapy. It suggests that some SA patients with relatively good lung functions might be undertreated.

The higher proportion of systemic corticosteroid dependence of SA patients with salicylate intolerance correlates with the result of a European survey, where systemic corticosteroid dependence was documented in 51% of asthmatic patients with salicylate intolerance^37^. The two sided association between systemic corticosteroid dependence and salicylate intolerance in SA patients in Table 3 is an interesting observation and has not yet been published elsewhere.

We think that these connections need to be confirmed in other SA populations and if they are consequent, it can help in treatment decisions and defining more exact disease endotypes.

## Conclusions

As a result of standardization of severe asthma definition, the percentage of severe asthma was 0.89% of all asthmatics in Hungary, which is lower than in other published studies. Using a clear study definition and a simple severe asthma questionnaire, reliable data were obtained from different areas of the country, which correlated with the data of a reference group with well-defined severe asthmatics and were similar to the results of international severe asthma consortia. Nevertheless, some data need further investigations as the occurrence of rhinosinusitis and salicylate intolerance and the rate of childhood onset asthma were significantly different between the two groups. In addition, as only 48% of the pulmonary dispensaries sent back the filled questionnaires, it could cause bias in our results. Using a novel Bayesian-based network analysis several interdependencies were revealed between patient characteristics. There was a strong direct positive casual relevance between salicylate intolerance and rhinosinusitis, best FEV1 value and rhinosinusitis and best FEV1 value and corticosteroid burst therapy. SA patients with rhinosinusitis had on average higher best FEV1 than patients without this comorbidity. Significantly more patients received regular oral systemic corticosteroid treatment who had salicylate intolerance, the proportion of rhinosinusitis was significantly higher in patients with salicylate intolerance and the proportion of salicylate intolerance was significantly higher in females. This analysis involving more characteristics can contribute to better endotyping of severe asthma which can help physicians in treatment decisions.

## Supplementary information

Supplementary information.

Supplementary information2.

## Data Availability

All data generated or analyzed during this study are included in this published article, and its supplementary information files.
