# Supplementary material for: Prevalence and characterization of severe asthma in Hungary
Source: Sci Rep. 2020 Jun 9;10:9274. doi: 10.1038/s41598-020-66445-4 (PMC7283249; doi:10.1038/s41598-020-66445-4)
Supplement: Supplementary file 1 — Supplementary information. [file 41598_2020_66445_MOESM1_ESM.docx]

| Social security number of the patient……………….………………. |
| --- |
| Name of the pulmonary outpatient clinic……………………………. |
| Name of the treating physician……………………….………………. |
| **Asthma characteristics** |
| Age of onset ……………years of age |
| Disease duration …………….year |
| Worst FEV1 during the treatment ……….L……….% |
| Best FEV1 during the treatment ……….L……….% |
| Allergic yes ̶ no |
| Smoking yes …………packs/year ̶ no |
| Per os corticosteroid is necessary continuously or during the larger part of the year yes (maintenance dose……….mg/day) ̶ no |
| In addition to the continuous ICS + LABA treatment per os corticosteroid burst therapy is necessary yes ̶ no |
| Salicylate intolerance yes ̶ no |
| Chronic rhinosinusitis, polyposis yes ̶ no |

**Supplementary table 1. Severe asthma database questionnaire**

Translated from the Hungarian questionnaire
